# Mesenchymal Stem Cells Lack Efficacy in the Treatment of Experimental Autoimmune Neuritis despite *In Vitro* Inhibition of T-Cell Proliferation

**DOI:** 10.1371/journal.pone.0030708

**Published:** 2012-02-16

**Authors:** Marija Sajic, David P. J. Hunt, Woojin Lee, D. Alastair S. Compston, Judith V. Schweimer, Norman A. Gregson, Siddharthan Chandran, Kenneth J. Smith

**Affiliations:** 1 Department of Neuroinflammation, Institute of Neurology, University College London, London, United Kingdom; 2 Department of Clinical Neurosciences, University of Cambridge, Cambridge, United Kingdom; 3 Department of Neuroimmunology, King's College London, London, United Kingdom; 4 MRC Centre for Regenerative Medicine, University of Edinburgh, Edinburgh, United Kingdom; Institute Biomedical Research August Pi Sunyer (IDIBAPS) - Hospital Clinic of Barcelona, Spain

## Abstract

Mesenchymal stem cells have been demonstrated to ameliorate experimental autoimmune encephalomyelitis (EAE), a model of multiple sclerosis, prompting clinical trials in multiple sclerosis which are currently ongoing. An important question is whether this therapeutic effect generalises to other autoimmune neurological diseases. We performed two trials of efficacy of MSCs in experimental autoimmune neuritis (EAN) in Lewis (LEW/Han ^M^Hsd) rats, a model of human autoimmune inflammatory neuropathies. No differences between the groups were found in clinical, histological or electrophysiological outcome measures. This was despite the ability of mesenchymal stem cells to inhibit proliferation of CD4+ T-cells *in vitro*. Therefore the efficacy of MSCs observed in autoimmune CNS demyelination models do not necessarily generalise to the treatment of other forms of neurological autoimmunity.

## Introduction

Therapeutic efficacy of early administration of high dose of bone marrow-derived mesenchymal stem cells (BM-MSCs) has been reported in the treatment of mouse experimental autoimmune encephalomyelitis (EAE), a model of multiple sclerosis (MS) [1], [2]. Clinical trials based upon the data are now underway in patients with MS [3], [4]. Previous studies suggested that these and other types of stem cells ameliorate autoimmune-mediated demyelination in the CNS through regulation of the inflammatory response [2], [5]. These findings raise an important question as to whether BM-MSCs might be beneficial in other inflammatory demyelinating diseases, such as Guillain-Barré syndrome (GBS) and chronic inflammatory demyelinating polyneuropathy (CIDP). Here, we tested whether intravenously delivered BM-MSCs were therapeutically beneficial in experimental autoimmune neuritis (EAN), an autoimmune peripheral neuropathy that has served as a model for GBS and CIDP [6], [7].

## Materials and Methods

### Experiment 1: Rat MSC characterisation and T-cell inhibition assays

Characterised, CD90+/CD45−/CD11b− Lewis rat bone marrow-derived mesenchymal stem cells (BM-MSCs) were obtained from Dr Darwin Prockop (kindly provided by the Tulane Center for Gene Therapy through a grant from NCRR of the NIH, Grant # P40RR017447). Their mesenchymal potential was confirmed with standard adipogenic and osteogenic assays [8], [9].

The ability of MSCs to inhibit T-cell proliferation *in vitro* was assessed with coculture studies using a myelin oligodendrocyte glycoprotein-reactive CD4^+^ rat T-cell line [10].

CD4+ lymphocytes were loaded with the cell division tracking dye carboxyfluorescein diacetate succinimidyl ester (CFSE Vybrant V-12883, Molecular Probes). Coculture studies were established in 12 well tissue culture plates in RPMI 1640 medium (Sigma-Aldrich R0883) with 10% fetal calf serum. MSCs were plated at a density of 100000 cells per well the day prior to coculture to achieve adherent MSC monolayer culture. CD4+ MOG-reactive T-cells were cocultured with either no MSCs, 1∶1 ratio of CD4+ cells:rat MSCs or 1∶1 ratio of CD4+cells: human MSCs. Cells were either unstimulated or stimulated with recombinant MOG and rat APCs as previously described [10]. After 48 hours of coculture CFSE staining in T-cells was analyzed by a FACScan flow cytometer (Dako). Proliferation index (defined as the sum of the cells in all generations divided by the computed number of parent cells) were calculated using Modfit LT 3.0 (Verity Software) [11]

### EAN Induction and Treatment Protocol

All experiments were licensed under the Animals (Scientific Procedures) Act 1986 of the UK Home Office. UK Home Office Project Licence Number: PPL 70/06710. EAN was induced in Lewis rats, strain LEW/Han ^M^Hsd (180–200 g; n = 50) by immunisation with peripheral bovine myelin and complete Freund's adjuvant (1 mg/ml *Mycobacterium tuberculosis*) [6].

Cells were harvested at passage 9 and resuspended in 1 ml Hanks Buffered Saline Solution (HBSS) at a concentration of 15×10^6^ cells/kg/ml. Animals were randomly assigned into four groups (n = 10 per group) to receive one of the treatments: i) BM-MSCs, ii) dermal fibroblasts (obtained by plating Lewis rat-derived dermal explants onto plastic in serum-containing media, and included as a cellular control), iii) dead BM-MSCs (killed by repeated freeze- thaw cycle), or iv) HBSS alone. A further, fifth control group of treatment-naïve animals (i.e. no injections) was also included. The treatments were administered under general anaesthesia as a slow intravenous injection five days after immunisation in a total volume of 1 ml: all cellular treatments were at a dose of 15×10^6^ cells/kg, in a blinded fashion. In separate cell tracing experiments, BM-MSCs were labelled with CFSE using identical cell dosages and treatment protocols to the main experiment.

### EAN scoring – primary and secondary outcome measures

The severity of neurological deficit in EAN was assessed by a blinded examiner on a ten point scale [6]. Immediately prior to the termination of the experiment (day 30), a measure of the number of functioning axons in the spinal roots and sciatic nerve was obtained electrophysiologically *in vivo*. The electrophysiological findings were assessed by a blinded examiner on a five point scale, where increasing score reflected greater amplitude and increasing complexity of the compound action potential, indicating increasing numbers of functional axons. The sciatic nerves were removed and one nerve was post-fixed in Karnovsky's solution and processed into resin for high resolution microscopy, and the other nerve was post-fixed in 3% paraformaldehyde solution, embedded in OCT and 12 µm thick longitudinal sections were labelled with anti-ED1 antibodies (Serotec,1∶2000; a marker of activated macrophages). The percentage of myelinated vs. demyelinated axons was quantified in ten randomly chosen fields within 1 µm toluidine-blue stained resin cross sections of sciatic nerves (100% represented a field where no axons were demyelinated). The number of ED1^+^ cells was determined by counting the cells in ten randomly chosen high power fields. In cell tracing experiments, lymphoid organs and peripheral nerve were removed at day 3 and 7 post BM-MSC injection, fixed in 3% paraformaldehyde and processed as described above.

### Experiment 2: Human MSC characterisation

Highly characterised human MSCs were obtained from Poietics and their *in vitro* phenotype (CD90+/CD105+/CD34−/CD45−) and mesenchymal differentiation confirmed using standard protocols [8], [9]. Inhibition of rat T-cell proliferation was confirmed using the same T-cell proliferation assay as described above.

### Treatment of EAN with human MSCs

Human MSCs were harvested at passage 9, suspended in HBSS at a concentration of 25×10^6^ MSCs per kg and injected day 7 post immunisation (n = 10). HBSS alone (no cells) was used as a control (n = 10). Immunisation and scoring was performed as previously described. Sciatic nerves were processed for immunohistochemistry as described above. 12 µm thick longitudinal sections were labelled with anti-human antibody human nuclear antigen (1∶100; Chemicon).

### Statistical analysis

Analysis of clinical and secondary EAN trial outcomes was undertaken using one way ANOVA and Kruskal-Wallis test.

## Results

### Functional characterisation of rat bone marrow-derived mesenchymal stem cells (i) Inhibition of T-cell proliferation

Lewis rat-derived CD90+/CD45−/CD11b− mesenchymal stem cells grew in monolayer culture and their ability to inhibit T-cell proliferation was assessed by coculture with myelin-reactive CD4+ T-cells.

**Figure 1:**
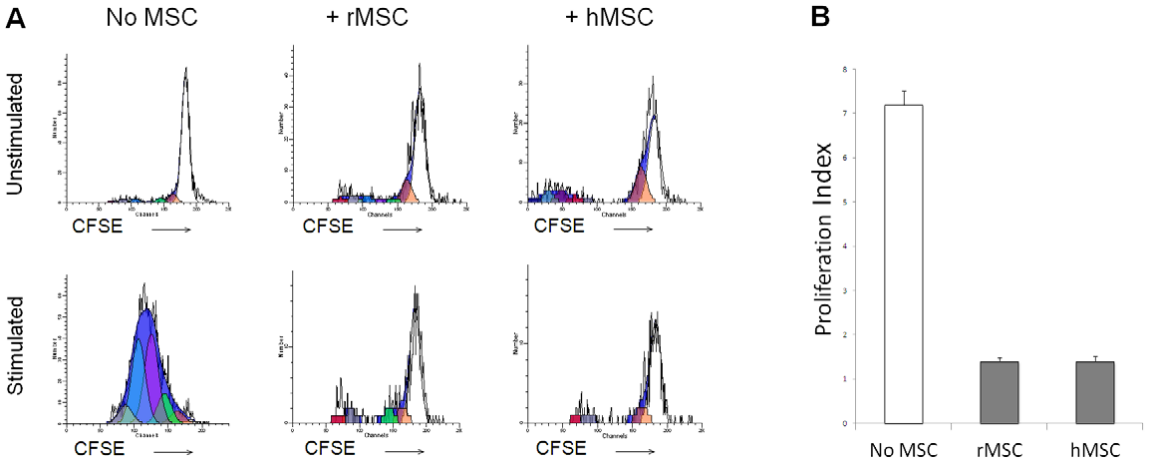
Mesenchymal stem cells derived from rat and human inhibit proliferation of myelin-reactive CD4+ T-cells *in vitro*. (A) CFSE proliferation assays, gated on CD4+ myelin reactive T-cells. Addition of a monoclonal myelin stimulus (MOG) leads to proliferation of T-cells, which is abolished by 1∶1 coculture with rat MSCs (rMSCs) and human MSCs (hMSCs). Representative FACS plots shown. (B) Coculture with rat mesenchymal stem cells and human mesenchymal stem cells leads to reduction in proliferation index of MOG-reactive CD4+ cells. (7.19+/−0.3 in absence of MSCs (n = 5), 1.4+/−0.1 with rat MSC coculture (n = 3), 1.4+/−0.1 with human MSC coculture (n = 3).

CFSE proliferation assays of rMOG-stimulated CD4+ T cells demonstrated that addition of mesenchymal stem cells (both rat and human in a 1∶1 ratio with CD4+ cells) reduced the proliferation index of CD4+ cells (Figure 1A, representative FACS plots). The proliferation index of MOG-stimulated CD4+ cells was 7.19+/− 0.3 (n = 5, figure 1B) in the absence of MSCs. The addition of Lewis rat MSCs reduced the proliferation index to 1.4+/−0.1 (n = 3, figure 1B). The addition of human MSCs to the coculture also reduced the proliferation index to 1.4+/−0.1 (n = 3, figure 1B). This effect was dose-dependent (not shown).

### Functional characterisation of rat bone marrow-derived mesenchymal stem cells (ii) Mesenchymal differentiation

**Figure 2:**
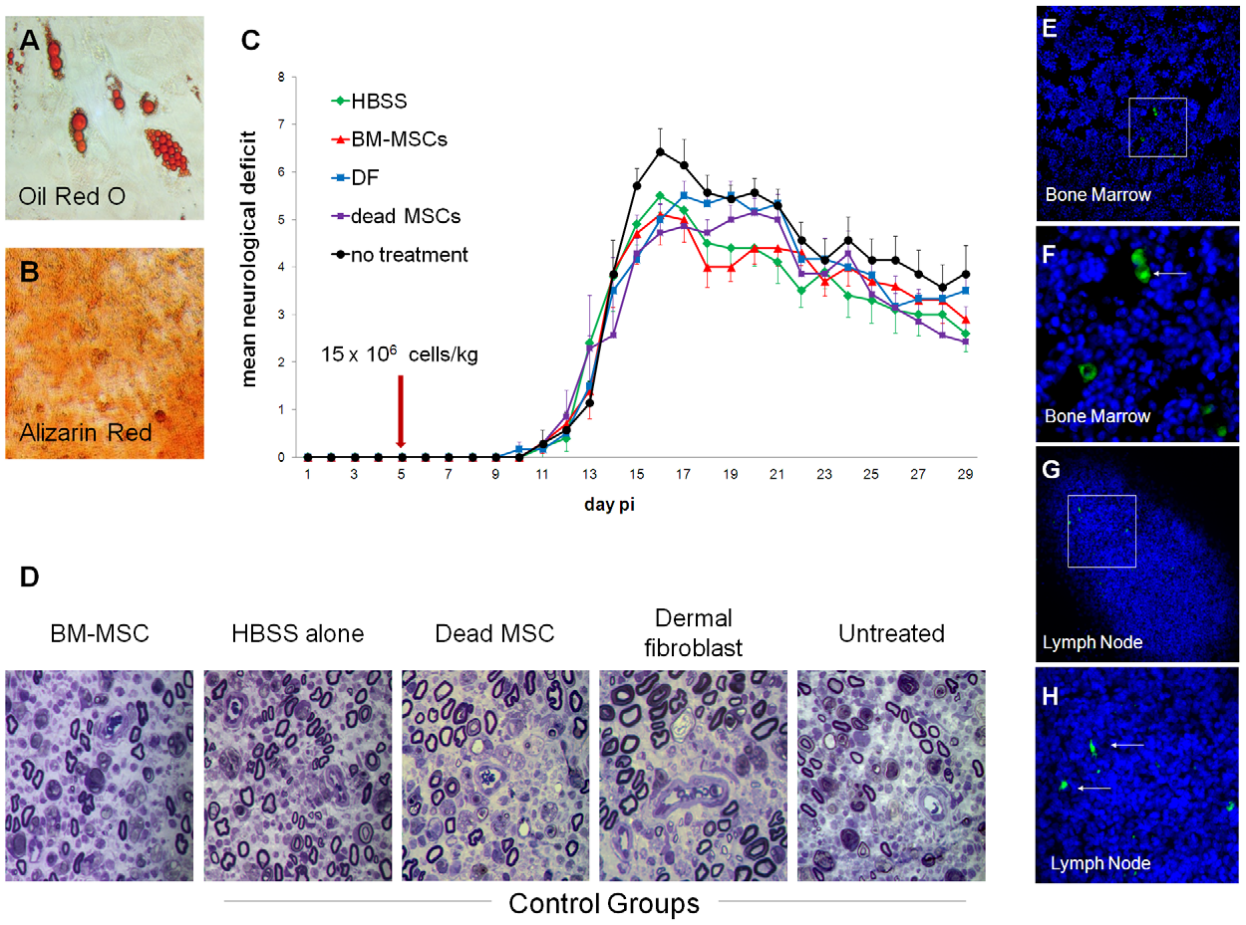
Lewis rat-derived MSCs do not improve clinical, electrophysiological or histological outcomes in experimental autoimmune neuritis. (A, B) Apidogenic and osteogenic potential of CD90+/CD45−/CD11b− Lewis BM-MSCs. Adipogenesis confirmed by Oil red O staining and osteogenesis confirmed by Alizarin Red staining. (C) Clinical course of EAN following various treatments. HBSS = Hank's buffered saline solution vehicle alone, BM-MSCs = 15×10^6^ bone marrow mesenchymal stem cells/kg, DF = 15×10^6^ dermal fibroblasts/kg, dead MSCs = 15×10^6^ dead bone marrow mesenchymal stem cells/kg, no treatment = no injection or anaesthesia. No significant differences in disease onset, severity or residual deficit were observed between groups. (D) Representative semithin sections from experimental groups. Inflammatory demyelination and axonal loss were observed in all groups. No histo-pathological differences were found between the treatment groups, as analysed in toluidine blue-stained micrographs of transverse sections (1 µm) through the sciatic nerves from animals in each group. (E–H) In cell tracing experiments, CFSE-labelled BM-MSCs were identified in bone marrow and lymphoid organs seven days after cell delivery (Hoechst – Blue; CFSE - Green; F & H show detail from E & G).

Lewis rat-derived MSCs differentiated appropriately into adipocytes and osteocytes. Functional differentiation was confirmed by oil red O staining and alizarin red staining, respectively (Fig. 2 A–B).

### No evidence of efficacy of rat MSCs in the treatment of EAN

Next, we treated animals with EAN by administrating BM-MSCs or control treatments. No adverse effects related to cell administration were observed in any of the treated animals. No significant differences between the groups were found with respect to time of disease onset (p = 0.76, Kruskal-Wallis test), peak severity of disease (p = 0.3, Kruskal-Wallis test), overall disease severity or magnitude of residual neurological deficit (p = 0.7 and p = 0.41, respectively, Kruskal-Wallis test and one-way ANOVA) (Table 1, Figure 2C). Furthermore, no significant differences were observed in a range of electrophysiological measures of axonal conduction resulting from stimulation of the spinal roots or sciatic nerves undertaken at the termination of the experiment (p = 0.88, Kruskal-Wallis test. Table 1).

**Table 1:** Outcome measures between BM-MSC treatment groups and control groups.

	BM-MSCs	HBSS alone	Dermalfibroblasts	DeadBM-MSCs	No treatment	Significance (p)
Median day of disease onset (range)	**12** (10–16)	**12** (10–13)	**12** (9–13)	**12.5** (10–14)	**12** (10–13)	0.76*
Median peak motor score (range)	**5.5** (4–7)	**5.5** (4–7)	**5** (4–6)	**5** (4–6)	**6** (4–9)	0.3*
Median terminal motor score (range)	**3** (0–5)	**3** (0–4)	**3.5** (2–5)	**2** (0–5)	**4** (2–6)	0.41*
Mean cumulative motor score (SD)	**65.3** (18)	**64.3** (17.6)	**69.6** (13.3)	**64.9** (21.9)	**76.9** (12.4)	0.7§
Electrophysiology: ankle-foot median response score (range)	**2/1** (0–4)/(0–4)	**1/2** (0–3)/(0–4)	**1/2** (0–3)/(0–4)	**2/2** (0–4)/(0–2)	**0/2** (0–2)/(0–3)	0.88*
Mean count of ED1^+^ cells per HPF (SD)	**194** (81)	**192** (68)	**199** (84)	**204** (86)	**238** (63)	0.71§
Mean % of myelinated axons per HPF (SD)	**25** (27)	**27** (20)	**27** (22)	**31** (24)	**9** (5)	0.34§

SD – standard deviation;

* Kruskal-Wallis test;

§ one way ANOVA, Tukey post-test;

HPF – high power field;

Similarly, quantitative assessment of demyelination (Figure 2D, Table 1) and macrophage infiltration of sciatic nerves (Table 1) showed no significant differences between the groups (p = 0.34, p = 0.71 respectively, one-way ANOVA).

Labelled BM-MSCs were histologically detected in bone marrow, lungs, spleen and lymph nodes, but not peripheral nerves, three and seven days post-administration (Figure 2E–H).

### Characterisation of human bone marrow-derived mesenchymal stem cells

**Figure 3:**
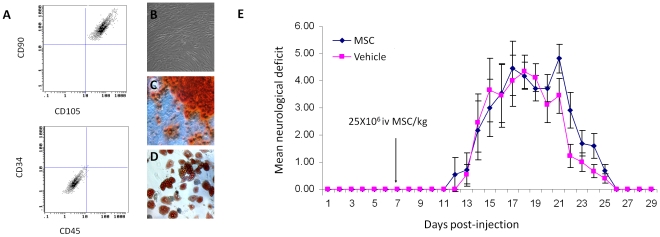
Human MSCs do not exert clinical effect in experimental autoimmune neuritis. (A) FACS profile of human MSCs confirms a CD90+/CD105+/CD34−/CD45− phenotype. (B) Human MSCs grow as confluent monolayers on tissue culture plastic and display mesenchymal differentiation (C) Osteogenesis: Alizarin red = orange, (D) Adipogenesis: Oil red O = red. (E) Human MSCs, delivered day 7 post immunisation at a dose of 25×10^6^ MSC/kg in 1 ml HBSS do not lead to a significantly different clinical outcome when compared with HBSS injection alone.

Human bone marrow-derived MSCs grew as confluent plastic adherent monolayers and FACS analysis confirmed the following cell surface marker phenotype: CD90+/CD105+/CD34−/CD45− (figure 3A). Human MSCs differentiated into adipocytes, osteocytes and chondrocytes in response to standard differentiation protocols (figure 3B,C and D) and inhibited rat T-cell proliferation to monoclonal stimuli. (figure 1A).

### No evidence of efficacy of human MSCs in the treatment of EAN

A second trial using human MSCs in the treatment of EAN was performed. A higher dose of 25×10^6^ cells/kg delivered at a later timepoint (day 7 post-immunisation). No significant difference was observed between MSC-treated and vehicle-only groups in both peak or cumulative disease scores (no significant difference, Kruskal-Wallis test, Figure 2E). No evidence of human mesenchymal stem cell engraftment in peripheral nerves was observed.

## Discussion

Previous reports have demonstrated a striking therapeutic efficacy of BM-MSCs when delivered early in the time course of EAE in mice [1], [2]. By contrast, we have shown that rat and human BM-MSCs, delivered intravenously at a similar time, do not lead to any discernable neurological, neurophysiological or histopathological improvement of EAN in Lewis rats, despite their mesenchymal potential and ability to inhibit T-cell proliferation *in vitro*.

It is unclear why rodent and human BM-MSCs have profound efficacy in EAE but lack any effect in EAN when delivered at a similar stage of disease. These findings may reflect interspecies differences in immune response to antigen and also the distinct nature of the respective antigens between central (EAE) and peripheral (EAN) nervous system components [1], [7]. It is thus possible that the immunomodulatory effect of BM-MSCs is specific to mouse EAE while lacking efficacy in other autoimmune neurological disorders with similar immunopathogenesis but different target antigens. Another explanation for the discrepancy between the effect of BM-MSCs in EAE in mice and EAN in rat, is the difference in cell doses used in the studies. We have used cell doses (per kg) at the lower end of those used in murine EAE studies. In published EAE and EAN trials the cell dosage is at least one order of magnitude higher than doses used routinely in clinical practice in humans [1], [2], [12], [13]. In the case of both rodent and human studies, an upper limit of cell dosage is placed by the limits of timing of culture and cellular load that can be safely delivered without adverse effects [13].

The *in vitro* inhibition of T-cell proliferation has been suggested to represent an *in vitro* model of the immunosuppressive properties of BM-MSCs [21], the proposed mechanism by which BM-MSCs ameliorate EAE [1], [2]. However, we and others have found that the *in vitro* immunosuppression is not specific to BM-MSCs [17]. For instance, we have found that a wide variety of plastic adherent cells, including dermal fibroblasts and an epithelial cell line derived from monkey kidney (Vero cells), exhibit such an effect (unpublished data). This cellular nonspecificity highlights the need to include cellular control groups (such as dermal fibroblasts) during *in vivo* trials.

Our experiments were primarily designed to see if MSC administration could prevent the development of EAN when given early in disease course. There is clear evidence of a gradient of response of experimental autoimmunity when treated with MSCs, suggesting that early treatment provides a maximal therapeutic response, which reduces significantly over time [2], [5]. For example in the treatment of EAE with MSCs there is only a very minor effect on scores of disease severity when MSCs are delivered at first clinical manifestation of disease, although earlier administration in the presymptomatic phase leads to very significant disease amelioration [2]. This time window for treatment is observed with many other immunosuppressive treatments in the context of experimental autoimmunity [20]. We therefore administered MSC treatment at two distinct timepoints during this early phase of immunopathogenesis where an immunosuppressive therapy might be anticipated to be maximally effective [19], [20]. Our experiments do not completely rule out the possibility that later (postsymptomatic) treatment with MSCs might lead to improvement by an alternative effects, such as engraftment of MSCs in the peripheral nerve, where they might mediate either neuroprotective or local anti-inflammatory effects. Such an immunomodulatory effect (within the target organ), has been observed in a model of EAE [18]. However, although we found evidence that MSCs migrated to peripheral lymphoid organs and bone marrow we found no evidence in either experiment to suggest that MSCs migrate to the peripheral nerve. It is therefore unlikely that MSCs exert a local immunosuppressive effect within the peripheral nerve.

It has been suggested that BM-MSCs may have a therapeutic role in a range of autoimmune diseases, including vasculitis, liver diseases, diabetes, inflammatory bowel disease and inflammatory neuropathies [14], [15], [16]. Our findings illustrate the importance of careful validation and experimental testing before the findings of pre-clinical studies in animal models are extrapolated to both larger species and different diseases, regardless of similarities in their immunopathogenesis.
